# Long term impact of ladder-related injuries as measured by the AQoL instrument

**DOI:** 10.1371/journal.pone.0235092

**Published:** 2020-06-23

**Authors:** Kym Roberts, Ogilvie Thom, Rob Eley, CJ. Cabilan, Kirsten Vallmuur

**Affiliations:** 1 Department of Emergency Medicine, Sunshine Coast Hospital and Health Service, Birtinya, Queensland, Australia; 2 Department of Emergency Medicine, Princess Alexandra Hospital, Woolloongabba, Queensland, Australia; 3 The University of Queensland, St Lucia, Queensland, Australia; 4 Australian Centre for Health Services Innovation, Queensland University of Technology, Brisbane, Queensland, Australia; 5 Jamieson Trauma Institute, Metro North Hospital and Health Service Royal, Brisbane and Women’s Hospital, Herston, Queensland, Australia; Federation University Australia, AUSTRALIA

## Abstract

**Introduction:**

Ladder-related falls are a common cause of patients presenting to emergency departments (ED) with serious injury. The impacts of ladder-related injuries were assessed at six-months post-injury using the quality of life, AQoL 4D Basic (AQoL) instrument.

**Materials and methods:**

This was a prospective observational study, conducted and reported according to the STROBE statement. All adult patients with ladder-related injuries who presented to two EDs in southeast Queensland, Australia between October 2015 and October 2016 were approached. Initial participant interviews took place at the time of ED presentation or shortly thereafter, with follow-up telephone interview at six-months.

**Results:**

There were 177 enrolments, 43 (24%) were lost to follow up. There were statistically significant changes post-injury for three of the four AQoL dimensions: independence, social relationships and psychological wellbeing, as well as the global AQoL. Twenty-four (18%) participants reported a clinically significant deterioration in independence, 26 (20%) participants reported a clinically significant deterioration in their social relationships, and 34 participants (40%) reporting a clinically significant deterioration in their psychological wellbeing. Nine of the twelve individual items (in AQoL dimension) deteriorated after injury, there was no change in two items (vision and hearing) and an improvement reported in one (communication). The largest changes (> 25% of participants) were reported with sleeping, anxiety worry and depression, and pain. Across the global AQoL dimension, 65 (49%) participants reported a clinically significant deterioration. The severity of injury as measured by the ISS was an independent predictor of the change in AQoL scores (p<0.001).

**Conclusions:**

Injuries related to falls from ladders continue to have a profound impact on patients at six-months post-injury as measured using the AQoL instrument. This adds to previous research which has demonstrated considerable morbidity and mortality at the time of injury.

**Prevention:**

Older males using ladders at home are at high risk for serious long-term injury. Injury prevention strategies and the safety instructions packaged with the ladder need to be targeted to this at-risk community group. There may also be a role for regulatory bodies to mandate a stabilising device to be included with the ladder at the time of purchase.

## Introduction

Ladder-related falls are a frequently preventable burden of injury. The rate of ladder-related falls is increasing with many patients presenting to emergency departments (EDs) with serious injuries [1, 2]. We have previously published a paper where a customised questionnaire to was used to establish the characteristics of the participants who experienced a ladder-related fall and the impact of these falls in the ED [1]. This study [1] found older adults who use ladders in non-occupational settings such as the home are the highest risk group for injury from a ladder fall which has been a similar finding in other studies [2, 3]. Studies have shown simple safety measures such as setting up and stabilising the ladder correctly, wearing appropriate footwear and not overreaching on the ladder are not being practiced in the home setting [1–3]. Previous research has focused on the incidence of these falls and immediate patient outcomes [2, 4–11]. A report on the long-term impact of ladder-related falls requiring admission to the intensive care unit (ICU) reported a profound effect with over half the survivors unable to live independently at 12 months post-injury [4]. However, that paper had two significant limitations to being representative of ladder falls; the majority of severely injured patients from ladder falls did not require admission to the ICU and those included in the study were likely to have a sustained severe traumatic brain injury [4].

The long-term impact of injuries is increasingly being examined from a patient’s perspective using quality of life (QoL) instruments [12]. However, there has been no published assessment of the long-term impact of ladder-related falls and sustained injuries assessing the patients’ QoL.

Our aim was to assess the impact of ladder-related injuries at six-months post-injury using the Assessment of Quality of Life (AQoL 4D Basic) instrument in all patients presenting to two EDs after a ladder-related fall over a 12-month period.

## Materials and methods

### Ethics

This study was approved by the Metro South Human Research Ethics Committee (HREC/15/QPAH/169). A participant information and consent form was given to the eligible participant in the ED, on the hospital ward or mailed out after initial contact by telephone if they had presented outside normal business hours and were discharged home after treatment. Written consent was obtained for the first interview and verbal consent was obtained and recorded for the follow-up interview. Informed consent was obtained from all individual participants in the study.

### Study design

This was a prospective observational study, conducted and reported according to the STROBE statement [13]. This is the second report from this project [1].

### Setting

The study was conducted within the Emergency Department, Princess Alexandra Hospital (PAH) and Department of Emergency Medicine, Nambour General Hospital (NGH), both located in southeast Queensland, Australia. PAH is a major tertiary referral hospital located in Brisbane which, at the time of the study, was receiving over 60,000 annual ED presentations. It has 24hour specialist trauma surgical services including neurosurgery and cardiothoracics. At the time of the study NGH was the major regional for the Sunshine Coast, receiving over 55,000 annual ED presentations and it did not have specialist neurosurgical and cardiothoracic services.

### Participants

Adult patients (18 years of age and over) who presented to the study EDs with injuries as a result of a fall from a ladder, or another object used as a substitute for a ladder were recruited to the study [1]. Exclusion criteria were participants being under the age of 18, unable to recall events, died in ED, or unable to access an interpreter [1]. The injuries sustained were classified as occupational or non-occupational by participants describing the place of injury (workplace versus home setting).

### Data collection

All adults presenting to PAH or NGH with injuries sustained from a ladder-related fall between October 2015 and October 2016 were approached to participate in the study. Recruitment of participants and data collection methods have been previously reported and results published [1]. Initial participant interviews took place at the time of ED presentation or shortly thereafter, establishing the circumstances of the ladder-related fall and also written completion of the AQoL 4D- Basic instrument. The results of this were used to establish the participants self-reported pre-injury health state prior to the incident occurring. A follow-up telephone interview took place six months post-injury and consisted of four questions, assessing the participants return to normal function or work, medical treatment, QoL changes, and reporting of product fault (ladder). At this time there was also a repeat of the AQoL instrument.

The use of AQoL 4D Basic has been validated for the purpose of comparing health states pre- and post-intervention [12, 14–16]. The AQoL 4D Basic is a descriptive four-dimensional self-assessment tool measuring a respondent’s health status; the dimensions measured are independence, social relationships, physical senses and psychological wellbeing [17]. The four dimensions are divided into three items/questions that are given a measurable value (utility score) to assess QoL as reported by the individual [17].

The characterisation of initial injuries for each participant was recorded from diagnoses reported in the Emergency Department Information System and the injuries grouped by anatomical region. Using this data, the injury severity score (ISS) was independently calculated by two investigators (KR and OT). Discrepancies were settled by consensus.

### Statistical analysis

Descriptive statistics were used to describe characteristics of the study population and analyse AQoL data. A paired t-test was utilised to analyse parametric data. The Wilcoxon signed-rank test was utilised to compare non-parametric paired data. The data was analysed using Stata 15.0 statistical software (Stata Corp, College Station, TX). A *p* value <0.05 was considered statistically significant.

As a baseline for comparison of change between pre- and post-injury health related QoL of participants the self-reported injury status tool AQoL 4D Basic was used [17]. To compare pre- and post-injury baseline scores the guidelines by Osoba et al [18] were followed. A change greater than 10% from baseline AQoL score at follow-up was deemed a clinically significant change [18]. The AQoL scores were converted to the utility measure using the STATA.do file available at http://aqol.com.au/index.php/scoring-algorithms?id=86, downloaded on 07/12/2017. A multiple regression model was built using the variables thought likely to independently predict change in the AQoL score. The variables in the model included hospital site, participant age, gender, participants pre-injury global AQoL score and the severity of their injury as determined by the ISS.

## Results

**Fig 1:**
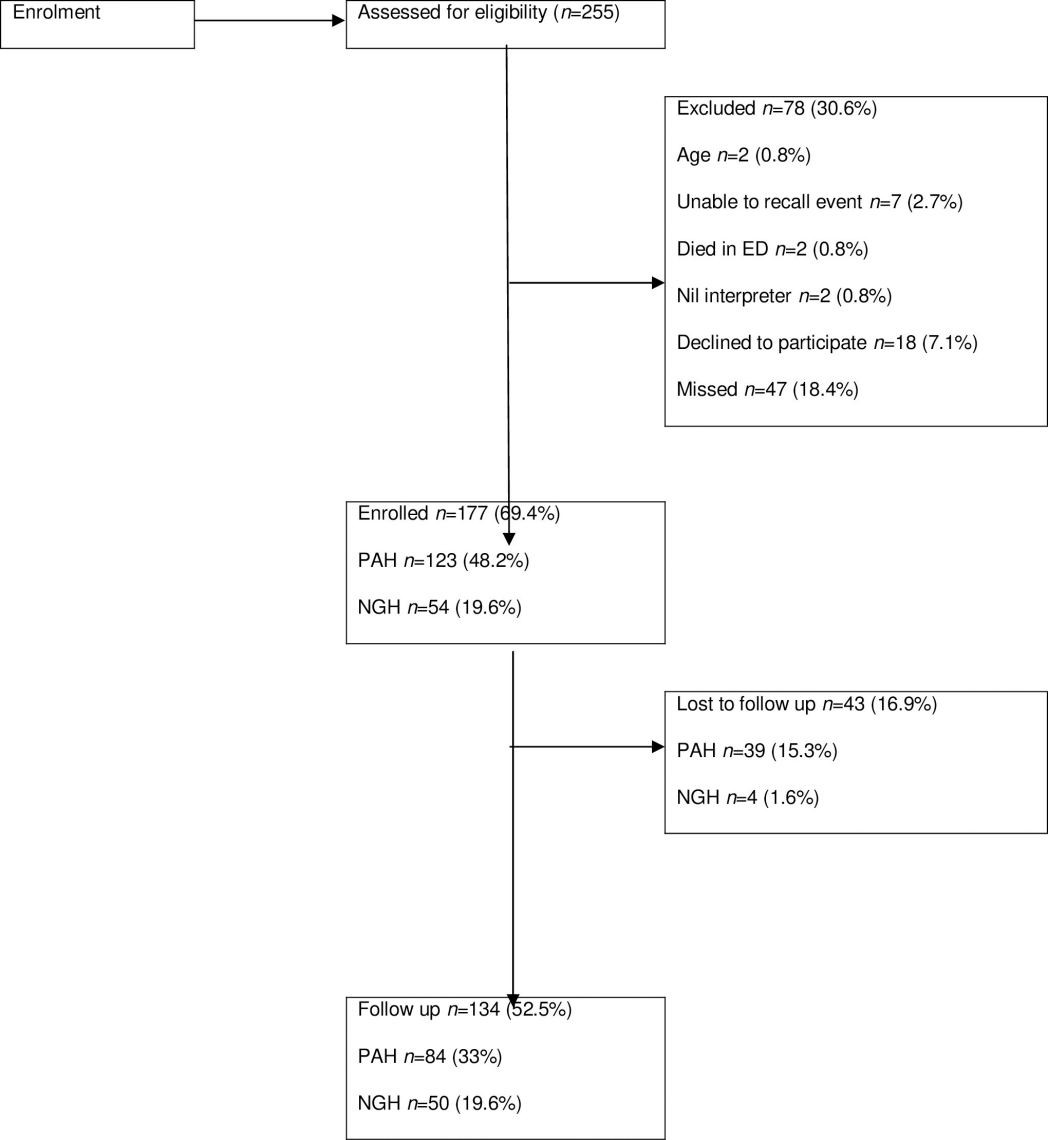
Participant flow diagram. ED, emergency department; PAH, Princess Alexandra Hospital; NGH, Nambour General Hospital.

There were 255 patient presentations from ladder-related falls between October 2015 to October 2016 to the study sites’ EDs; 185 from PAH and 70 from NGH (Fig 1) [1].

Of the 255 presentations, 13 were ineligible for recruitment, 18 declined to participate, and 47 presentations were missed (presented to ED outside working hours of research nurses and unable to contact the patient after three attempts by telephone post-discharge from ED) [1]. Of the remaining 177 participants, 43 (24%) were lost-to-follow-up, 39 (15.3%) from PAH and 4 (1.6%) from NGH. One hundred and thirty-four patients participated in the six-month follow up, 84 (33%) from PAH and 50 (19.6%) from NGH.

### Population characteristics

The characteristics of the study population at the six-month follow-up (F/UP) and those lost to follow-up (LT/FU) are presented in Table 1. The typical participant was male (81%), over 55 years of age (73%), had fallen from a height greater than one metre (86%) and injured in the non-occupational setting (71%). The LT/FU participants were younger (47 versus 57 years of age, *p* <0.001), less severely injured (ISS 4 versus 2, p = 0.032) and from PAH (*p* = 0.001). There were no other differences between the F/UP and LT/FU groups.

**Table 1:** Characteristics of the study population.

**Variables**	**F/UP** (*n* = 134) *n* (%)	**LT/FU** (*n* = 43) *n* (%)	**p value**
**Site**			
PAH	84 (63)	39 (91)	0.001
NGH	50 (37)	4 (9)	
**Age median (range)**	57 (18–87)	47 (19–83)	<0.001
**Age groups (years)**			
18–29	7 (5)	9 (20)	0.80
30–39	15 (11)	6 (14)	0.07
40–49	15 (11)	9 (21)	0.54
50–59	28 (21)	8 (18)	0.001
60–69	36 (27)	6 (14)	<0.001
70–79	27 (20)	2 (5)	<0.001
80–89	6 (5)	3 (7)	0.51
**Sex**			
Male	109 (81)	36 (84)	0.72
Female	25 (19)	7 (16)	
**Fall height > 1metre**	116 (66)	41 (23)	0.39
**Place of Injury**			
** Home**	95 (70)	27 (64)	0.27
** Workplace**	40 (30)	15 (36)	
**ISS Median (IQR)**	4 (1–5)	2 (1–4)	0.032
**ISS Range**	0–22	1–25	
**Disposition**			
Hospital admission	50 (37)	13 (30)	0.40
**HLOS days median (IQR)**	5 (3–11.5)	2 (1–7)	0.21

PAH, Princess Alexandra Hospital; NGH, Nambour General Hospital; F/UP, followed up; LT/FU, lost to follow up; ISS, Injury Severity Score; HLOS, hospital length of stay; and IQR, interquartile range.

### Place of injury

Participants more commonly experienced a ladder-related fall at home, outdoors using a ladder (n = 95/122, 78%) rather than indoors (n = 27/122, 22%) (see Table 2). At home the most common type of activity resulting in a ladder-related fall was home maintenance; this was true for both inside and outside the home. The second most common activity involving a ladder-related fall was pruning.

**Table 2:** Activity at time of injury where place of injury = Home.

Variables	F/UP	LT/FU
**Home group** *n* = 122	*n* = 95	*n* = 27
**Indoor**	*n* = 22/95	*n* = 5/27
Accessing/retrieving	7	2
Cleaning	1	0
Home maintenance	14	3
**Outdoor**	*n* = 73/95	*n* = 22/27
Accessing/retrieving	2	0
Cleaning	4	1
Gardening	4	0
Pruning	22	7
Home maintenance	36	13
Other maintenance (e.g. caravan)	2	0
Other miscellaneous	3	1

F/UP, followed up; LT/FU, lost to follow-up

In the workplace (presented in Table 3) participants more commonly experienced a ladder-related fall whilst performing their occupational task (60%), rather than while ascending or descending the ladder (40%). Occupational tasks included, for example, a carpenter fixing ceiling fascia, an electrician installing a cable, and a maintenance worker painting a roof gutter.

**Table 3:** Activity and occupation at time of injury where place of injury = Work.

	L/FU (*n* = 40)		LT/FU (*n* = 15)	
Occupation	Ascending or descending	Performing occupation on ladder	Ascending or descending	Performing occupation on ladder
Builder	6 (15%)	5 (13%)	1 (7%)	2 (13%)
Carpenter	0	4 (10%)	1 (7%)	1 (7%)
Electrician	1 (3%)	3 (8%)	0	0
Maintenance	0	4 (10%)	0	2 (13%)
Painter	2 (5%)	3 (8%)	4 (27%)	0
Refrigeration technician	1 (3%)	0	1 (7%)	0
Retail assistant	0	1 (3%)	1 (7%)	1 (7%)
Others	4 (10%)	6 (15%)	0	1 (7%)
**Total**	14 (35%)	26 (65%)	8 (53%)	7 (47%)

F/UP, followed up; LT/FU, lost to follow-up

### Injuries

There were numerous injuries (*n* = 277) sustained by participants (presented in Table 4) as a result of ladder-related falls. Sixty-eight (38%) participants suffered injuries to more than two body parts. The most common location of injury encountered by participants included spinal fractures (*n* = 31), rib fractures (*n* = 20), tibia/fibula fractures (*n* = 15), radius/ulna fractures (n = 14), pelvic fractures (*n* = 10), and traumatic pneumothorax (*n* = 8). The ISS ranged from zero to 25, with four patients meeting the definition of major trauma (ISS > 15). One of the major trauma patients was in the lost to follow up group.

**Table 4:** Location and nature of injuries.

Location (*n* = 277)	F/UP (*n* = 222) *n* (%)	LT/FU (*n* = 55) *n* (%)
**Head injuries** (*n* = 8)		
Retrograde amnesia	2 (0.7)	
Concussion	2 (0.7)	
Fracture	2 (0.7)	
Subarachnoid haemorrhage		1 (0.4)
Closed head injury	1 (0.4)	
**Spinal injuries** (*n* = 31)		
Fracture	27 (9.7)	3 (1.1)
Spinal cord injury	1 (0.4)	
**Thoracic injuries** (*n* = 37)		
Rib fractures	18 (6.5)	2 (0.7)
Pneumo/haemothorax	6 (2.2)	2 (0.7)
Clavicle fracture	4 (1.4)	
Pulmonary contusion	1 (0.4)	2 (0.7)
Scapula fracture	2 (0.7)	
**Abdominal/pelvic injuries** (*n* = 10)		
Pelvic fracture	9 (3.2)	1 (0.4)
**Upper limb injuries** (*n* = 31)		
Humerus fracture	2 (0.7)	2 (0.7)
Radius/ulna fracture	12 (4.3)	2 (0.7)
Dislocations	4 (1.4)	
Other fractures	6 (2.2)	3 (1.1)
**Lower limb injuries** (*n* = 36)		
Femur fracture	2 (0.7)	
Neck of femur fracture		1 (0.4)
Tibia/fibula fracture	15 (5.4)	5 (1.8)
Calcaneal fracture	5 (1.8)	3 (1.1)
Other fractures	4 (1.4)	1 (0.4)
**Other injuries** (*n* = 125)		
Abrasions	13 (4.7)	2 (0.7)
Contusion	15 (5.4)	6 (2.2)
Haematoma	12 (4.3)	3 (1.1)
Lacerations	26 (9.4)	6 (2.2)
Pain	28 (10.1)	8 (2.9)
Miscellaneous	6 (2.2)	

Miscellaneous injuries: tendon injuries, electrocution, paraesthesia and vertigo. F/UP, followed up; LT/FU, lost to follow-up

The hospital length of stay (HLOS) varied greatly among participants, median 5 days, IQR 3–11.5, (range 1–192).

### Return to work

Twenty-eight (21%) of the 134 F/UP participants had returned to work (presented in Table 5) within one week of their injury. However, after six-months post-injury 22 participants (16%) had either not returned to work or not fully recovered from their injuries. The 112 (84%) participants who had returned to work by the six-month interval did so after a median period of eight weeks (IQR 4–12).

**Table 5:** Return to work/normal function.

Age (years)	< 1 week *n* = 28(21%)	1–4 weeks *n* = 27(20%)	1–3 Months *n* = 40(30%)	4–6 Months *n* = 17(13%)	DNR *n* = 22(16%)	Total (*n* = 134)
18–29	1	3	3	0	0	7(5%)
30–39	2	3	4	5	1	15(11%)
40–49	4	4	3	1	3	15(11%)
50–59	9	1	7	6	5	28(21%)
60–69	7	7	14	3	5	36(27%)
70–79	5	5	8	1	8	27(20%)
80–89	0	4	1	1	0	6(5%)

DNR; did not return to work or normal function by six-months.

### Quality of life

The AQoL Basic items and dimensions are presented in Table 6. Nine of the twelve individual items deteriorated after the injury while there was no change in two (vision and hearing) and an improvement reported in one (communication). These three items contribute to the physical senses dimension. The largest changes (> 25% of participants) were reported in the sleeping item, the anxiety worry and depression item, and the pain item. All nine items that showed a deterioration after the injury had statistically significant changes. As a result, there were statistically significantly changes post-injury for three of the dimensions: independence, social relationships and psychological wellbeing, as well as the global AQoL. One participant declined to answer AQoL items 4 and 5 for social relationships.

**Table 6:** AQoL utility scores pre- and at six-months post-injury *n* = 134.

AQoL item	Improved n (%)	Unchanged n (%)	Deteriorated n (%)	**Pre-injury** Mean (±SD)	**Post-injury** Mean (±SD)	**Mean change** **(*P* value)**
1. Help dressing, bathing, eating	1 (<1)	124 (93)	9 (7)	1.03 (± 0.21)	1.14 (±0.54)	0.11 (0.011)
2. Help cooking, cleaning, washing	3 (2)	110 (82)	21 (16)	1.06 (±0.29)	1.26 (±0.67)	0.20 (<0.001)
3. Getting around home and community	0	118 (88)	16 (12)	1.01 (±0.12)	1.21(±0.60)	0.19 (<0.001)
4. Health impact on relationships in general*	6 (4)	114 (86)	13 (10)	1.17 (±0.58)	1.25 (±0.63)	0.08 (0.099)
5. Relationships with others*	5 (4)	113 (85)	15 (11)	1.19 (±0.55)	1.33 (±0.69)	0.14 (0.022)
6. Impact on relationships/role in family	7 (5)	108 (81)	19 (14)	1.10(±0.34)	1.23 (±0.56)	0.13 (0.016)
7. Impact on vision	5 (4)	121 (90)	8 (6)	1.19 (±0.43)	1.21 (±0.46)	0.02 (0.405)
8. Impact on hearing	6 (4)	125 (93)	3 (2)	1.19 (±0.43)	1.16 (±0.41)	-0.02 (0.317)
9. Communicating with others	6 (4)	128 (96)	0	1.07 (±0.29)	1.03 (±0.17)	-0.04 (0.014)
10. Sleeping	13 (10)	87 (65)	34 (25)	1.49 (±0.83)	1.84 (±1.06)	0.35 (<0.001)
11. Anxiety, worry, depression	13 (10)	88 (66)	33 (25)	1.31 (±0.58)	1.51 (±0.75)	0.21 (0.002)
12. Pain	3 (2)	87 (65)	44 (33)	1.26 (±0.50)	1.59 (±0.67)	0.33 (<0.001)
**AQoL Dimension**						
**Independence** (Items 1–3)	3 (2)	107 (80)	24 (18)	0.99 (±0.57)	0.93 (±0.19)	-0.06 (<0.001)
**Social relationships** (Items 4–6)*	14 (10)	90 (68)	29 (22)	0.95 (±0.14)	0.92 (±0.17)	-0.03 (0.015)
**Physical senses** (Items 7–9)	13 (10)	113 (84)	8 (6)	0.96 (±0.63)	0.97 (±0.05)	0.01 (0.239)
**Psychological wellbeing** (Items 10–12)	20 (15)	55 (41)	59 (44)	0.95 (±0.83)	0.89 (±0.15)	-0.6 (< 0.001)
**Global AQoL**	26 (19)	43 (32)	65 (49)	0.87 (±0.18)	0.77 (±0.27)	-0.10 (< 0.001)

AQoL, assessment of quality of life; SD, standard deviation

* one participant declined to answer two questions (AQoL items 4 & 5) for social relationships

As previously mentioned a clinically significant change has been determined to be a change of greater than 10% [18]. For the independence dimension 24 (18%) participants reported a clinically significant deterioration, for the social relationships dimension 26 (20%) participants reported a clinically significant deterioration, and psychological wellbeing dimension had 34 participants (40%) reporting a clinically significant deterioration. Two participants (2%) reported a clinically significant deterioration in the physical senses’ dimension. Across the global AQoL dimension, 65 (49%) participants reported a clinically significant deterioration.

Patients age, gender, and the pre-injury level of functioning (AQoL) did not predict the change in AQoL scores between pre and post-injury. The severity of injury as measured by the ISS was an independent predictor of the change in AQoL scores (p<0.001).

## Discussion

The typical patient who presents to the ED after a fall from a ladder has been well described [1]. They are male, aged over 50 years, have fallen at home, often from a height greater than one metre and stay in hospital for five days [1, 4–7, 9, 19–22]. This study found the most common activity being completed by participants at home at the time of the ladder fall was home maintenance and tree pruning. In contrast, falls from ladders in occupational settings typically involve younger patients and less frequent serious injury [7, 19]. The proportion of less significant injuries associated with occupational injury may be in part related to the requirement for medical certification due to state occupational health practices (such as Workcover).

This study demonstrates for the first time the significant long-term impact on QoL of falls from ladders. The AQoL dimension of independence focused on assessing the participants level of self-care, ability to perform household duties and mobility [14]. The results show almost 1 in 5 participants reported a loss of independence compared to their pre-injury level. Older adults (over the age of 65 years) who encounter serious injury as a result of a fall at home often experience a loss of independence, ability to perform activities of daily living and household duties, physical function and activity [23–26].

Social relationships in the AQoL instrument included self-assessment of friendship, isolation and family roles [14]. Our results reveal that participants experienced deterioration in their social relationships post-injury, consistent with published studies showing older adults who experience severe injury as a result of a fall will often limit their social activities [23, 26]. This is often due to the physical and psychological impact of injury which can cause an increased experience of fear, anxiety and depression in the post-injury period [23, 26].

The largest deterioration was in the psychological wellbeing dimension, participants experienced disruption to sleep patterns, increased pain and worrying post-injury. This is consistent with previous findings on psychological distress in adults with serious injury resulting from a fall [24, 25, 27]. Symptoms of psychological distress are not only encountered in the acute injury stage but can also persist well into the post-injury period [24]. This supports the need for health services to provide screening for trauma patients for symptoms of post-traumatic stress disorder during their hospital admission and also follow-up during the recovery period [24, 26, 27].

In the physical senses’ dimension participants reported no significant changes in their vision or hearing. There was however an improvement in the AQoL score for communication with others. We are not sure why this occurred, but one clue may come from one participant’s comment that he had used this opportunity to improve his healthy behaviours and become more active.

Our results indicate the severity of injury independently predicted the changes in the QoL measurements. The participants age, gender and pre-injury QoL status did not predict the post-injury changes. Increasing age has been found to be associated with higher ISS [4, 6, 19] and greater HLOS [4, 19] in previous studies on ladder injuries. However, we must note that our median ISS was lower than others have reported [4, 6, 19]. This may indicate an even stronger relationship exists between the ISS and change in AQoL post-injury than we have found.

It was hoped that as well as documenting the impact of their injuries, the AQoL would provide information that might allow for targeted intervention strategies after discharge from hospital for these patients. The multidimensional impact on QoL with clinically significant changes in independence, social relationships and psychological well-being suggest a ‘one size fits all approach’ is unlikely to be effective. These findings emphasise the complex relationship between injury and recovery, between functional status, disability and quality of life.

Due to the fact that we collected participants self-reported pre-injury AQoL scores at the time of presentation to the ED, it is possible that a bias may have been introduced as the AQoL scores were completed post-injury. The possibility of ‘floor effect’ bias in the pre-injury result also cannot be ignored. This could lead to an overestimation of the difference between pre and post injury scores. However, we feel our results were an accurate reflection of the participants’ pre-injury health status. Our pre-injury Global AQoL mean of 0.87 (see Table 6) exactly matches that of a normal Australian population reporting very good health [14]. It is higher than the population mean (0.79) and almost identical to the median (0.89) for males aged 50–59, our typical patient [14]. For convenience, AQoL scores were recorded face-to- face of by telephone at the initial interview and by telephone for the six-month follow-up interview. This may have introduced a methodological bias. However, the pre-injury AQoL was not predictive of the changes experienced by participants.

Added to the burden of injury itself is the time taken for the participant’s recovery. The results of our study revealed that 16% of participants had not either returned to work or not fully recovered by six-months. The median period of disability was eight weeks prior to the participant returning to work (light or full duties) or normal function. This disability period is greater than the previously reported average disability of six weeks post-injury from ladder falls [6, 20].

### Prevention

Our findings underscore the need for, and should inform, injury prevention strategies in the community. Community prevention and education programs should specifically target older males in the non-occupational setting. There is also clearly a role for community groups, retailers and regulatory authorities to improve the dissemination of safety devices (such as a stabilising device to be included in purchase) and instruction on safe handling techniques provided to purchasers of ladders.

Within the non-occupational setting there is a widespread lack of knowledge regarding use of safety equipment and methods to improve personal safety while using ladders in comparison to the occupational setting [3, 28]. A recent campaign run by the Australian Competition and Consumer Commission (ACCC) focused on older males in the community who are at a high risk of injury [8, 9, 29]. However, there has been no published evaluation of the recent ACCC campaign to assess the efficacy of the program and further work is needed to investigate the most appropriate methods for delivering safety messages to this target group.

In addition to improved safety designs by ladder manufacturers, retailers who sell ladders for non-occupational use need to incorporate increased safety awareness among retail staff to ensure safety advice is provided as part of the purchase, such as in the form of training, brochures or greater promotion of safety devices [4]. There are a number of devices that can be purchased that assist to secure the ladder while in use, including rubber feet, hooks, extender arms, fasteners and stabilisers [28]. Also there are methods to improve personal safety while using the ladder such as setting up and positioning the ladder correctly, adding non-slip rung covers, and wearing appropriate safety footwear [28] and potentially the use of helmets [3, 30]. This may provide a key opportunity to not only educate and increase safety awareness of ladder use among non-occupational users, but also prevent serious injury from ladder-related falls, particularly among older adults in the community.

As an alternative approach to be considered, specific education programs teaching when to avoid using ladders at home should be considered. Educating older adults to request help from others or employ home service contractors to complete tasks around the home that involve the use of a ladder is an important injury prevention strategy that should be specifically targeted to this group [21]. This may be crucial in preventing injury from ladder-related falls among this age group [9]. A falls prevention program that targeted older adults and provided education within their home about the risk of falls was successful in reducing falls in the home by 31% [9]. A similar program could be tailored to reducing ladder-related injuries.

### Study limitations

The small number of participants in our study compared with previous reports on ladder injuries [7, 9] reflects both the prospective nature of our study and our use of the AQoL instrument. We demonstrated similar mortality and hospital admission rates to these larger studies, suggesting our participants are similar. We also had a large number of participants lost to follow-up (18.4%) and missed patients (16.9%) in the recruitment process. This group was significantly younger than those completing the AQoL and largely from a single site. We initially postulated that this might be explained by a higher proportion of occupational falls (thus difficult to reach during normal working hours for the follow-up) or a lesser injury burden being reflected in a lower participation rate. The LTFU group did have a lower ISS (p = 0.032). Given the strong influence of the ISS on the changes to AQoL scores, it is possible the changes we found may over-estimate the long-term impact of all ladder injuries if the LTFU group had been included.

Another limitation of our study was that the ISS was calculated retrospectively by two authors. Both are senior emergency clinicians but have not been formally trained in ISS coding. We recorded lower ISS scores than previously reported from ladder-related injuries, which may weaken the relationship between ISS and change in AQoL scores.

## Conclusion

Injuries related to falls from ladders continue to have a profound impact on patients at six months post-injury as measured using the AQoL instrument. This adds to previous research demonstrating considerable morbidity and mortality at the time of injury. The spread of QoL dimensions affected indicates interventions targeted at improving QoL in affected patients will need to be individualised. Further research would be helpful in establishing the timeline of changes in QoL in ladder-related injuries as well as the proportion of patients whose QoL is affected permanently. These are frequently preventable injuries and our findings again demonstrate the need for preventative measures to be taken.

## Abbreviations

ED: Emergency Department
AQOL: Assessment Quality of Life
ISS: Injury Severity Score
QOL: Quality of Life
F/UP: Follow UP
LT/FU: Lost to Follow Up
HLOS: Hospital Length of Stay
ACCC: Australian Competition and Consumer Commission
